# Absence of CD34 on Murine Skeletal Muscle Satellite Cells Marks a Reversible State of Activation during Acute Injury

**DOI:** 10.1371/journal.pone.0010920

**Published:** 2010-06-02

**Authors:** Nicholas Ieronimakis, Gayathri Balasundaram, Sabrina Rainey, Kiran Srirangam, Zipora Yablonka-Reuveni, Morayma Reyes

**Affiliations:** 1 Department of Pathology, School of Medicine, University of Washington, Seattle, Washington, United States of America; 2 Department of Biological Structure, School of Medicine, University of Washington, Seattle, Washington, United States of America; McMaster University, Canada

## Abstract

**Background:**

Skeletal muscle satellite cells are myogenic progenitors that reside on myofiber surface beneath the basal lamina. In recent years satellite cells have been identified and isolated based on their expression of CD34, a sialomucin surface receptor traditionally used as a marker of hematopoietic stem cells. Interestingly, a minority of satellite cells lacking CD34 has been described.

**Methodology/Principal Findings:**

In order to elucidate the relationship between CD34+ and CD34- satellite cells we utilized fluorescence-activated cell sorting (FACS) to isolate each population for molecular analysis, culture and transplantation studies. Here we show that unless used in combination with α7 integrin, CD34 alone is inadequate for purifying satellite cells. Furthermore, the absence of CD34 marks a reversible state of activation dependent on muscle injury.

**Conclusions/Significance:**

Following acute injury CD34- cells become the major myogenic population whereas the percentage of CD34+ cells remains constant. In turn activated CD34- cells can reverse their activation to maintain the pool of CD34+ reserve cells. Such activation switching and maintenance of reserve pool suggests the satellite cell compartment is tightly regulated during muscle regeneration.

## Introduction

Since their discovery, satellite cells have been characterized as the resident stem cells of the skeletal muscle, responsible for postnatal myofiber growth and regeneration [1], [2]. Classically, satellite cells have been defined by their position underneath the basal lamina of muscle fibers. More recently, satellite cells have been distinguished by nuclear *Pax7* immunostaining and/or *Myf5^nlacZ/+^* reporter expression, and the presence of various surface receptors including α7 integrin (herein referred as α7), β1 integrin, CD34, NCAM, c-met, and CXCR4 [3], [4], [5], [6], [7], [8], [9], [10], [11], [12]. Although satellite cells are unanimously recognized by anatomical location, there is no single surface marker specific or exclusive to the entire population. This issue is compounded by the heterogeneity of satellite cells between muscles, as reported with *Pax3* expression, and within muscles, based on non-uniform expression levels of *Myf5*-driven reporters [13], [14], [15]. Such heterogeneity between and within various muscles exemplifies the complexity of the satellite cell pool that has yet an established canonical differentiation lineage.

Beauchamp et al. (2000) reported that the majority of satellite cells could be identified by *Myf5-*knock-in reporter activity and CD34 expression. In this study approximately 20% of satellite cells monitored in freshly isolated myofibers from extensor digitorum longus (EDL) muscle, could not be detected by CD34 immunostaining. Although the CD34- satellite cell population was not characterized, it was suggested these cells may represent a more primitive population of muscle stem cells [5]. In separate studies using *Pax3* and *Pax7* reporter mice, it was observed that the majority of myogenic cells of skeletal muscle reside within the CD34+ fraction [16], [17]. It has been further described that myogenic cells can be isolated by fluorescence-activated cell sorting (FACS) as CD45−/Sca1−/CD34+, and more recently as CD45−/CD31−/Sca1−/CD11b−/α7+/CD34+ [18], [19].

Although CD34 has been used to identify and isolate satellite cells, many cell types, including muscle endothelial cells, express CD34 [20]. Thus, in order to study satellite cell heterogeneity, we initially set out to develop a method to isolate pure populations of satellite cells using the CD34 antigen while excluding other cells that may also express CD34. Utilizing FACS based on previously published reports from this and other laboratories, we initially isolated satellite cells by removing CD45+ hematopoietic cells, CD31+ endothelial cells, other non-satellite Sca1+ cells, and then selecting the remaining CD34+ fraction [18], [20], [21]. Although the bulk of CD45−/CD31−/Sca1−/CD34+ sorted cells were myogenic, we repeatedly observed non-myogenic cells in culture. Next, in accordance with the literature, we incorporated α7 for satellite cell FACS isolation [10], [14], [19], [22]. Indeed, this approach eliminated non-myogenic cells in culture. Subsequently, we observed by FACS-analysis that the CD45−/CD31−/Sca1−/α7+ population was comprised of a CD34+ majority and CD34− minority. In the murine hematopoietic system it has been observed that single CD34−/low stem cells can reconstitute the entire hematopoietic system and give rise to both CD34+/− cells [23]. Therefore, in analogy with the hematopoietic system, we initially hypothesized that within the satellite cell compartment the minority of CD34− cells may represent more primitive cells upstream of the more abundant CD34+ population. However, our results described herein indicate the absence of CD34 on skeletal muscle satellite cells *in vivo* marks a state of activation dependent on muscle injury and not necessarily a hierarchy as initially predicted.

## Results

### Despite CD45/CD31/Sca1 Negative Selection, CD34 is Insufficient for Purifying Satellite Cell by Flow Cytometry

Using our previously developed method to isolate endothelial cells from a collection of skeletal muscles (pooled hind limb, pectorals and triceps muscles), we initially FACS-sorted satellite cells as CD45−/CD31−/Sca1−/CD34+ [20]. Reverse-transcription PCR (RT-PCR) of these freshly sorted cells revealed expression of *Pax7* and myogenic factors *Myf5* and *MyoD* (data not shown). In culture, the majority of CD45−/CD31−/Sca1−/CD34+ sorted cells produced colonies comprised of small round cells that ultimately formed twitching myotubes. However, cultured CD45−/CD31−/Sca1−/CD34+ cells also gave rise to larger non-myogenic cells that never formed myotubes (data not shown)**.**

**Figure 1:**
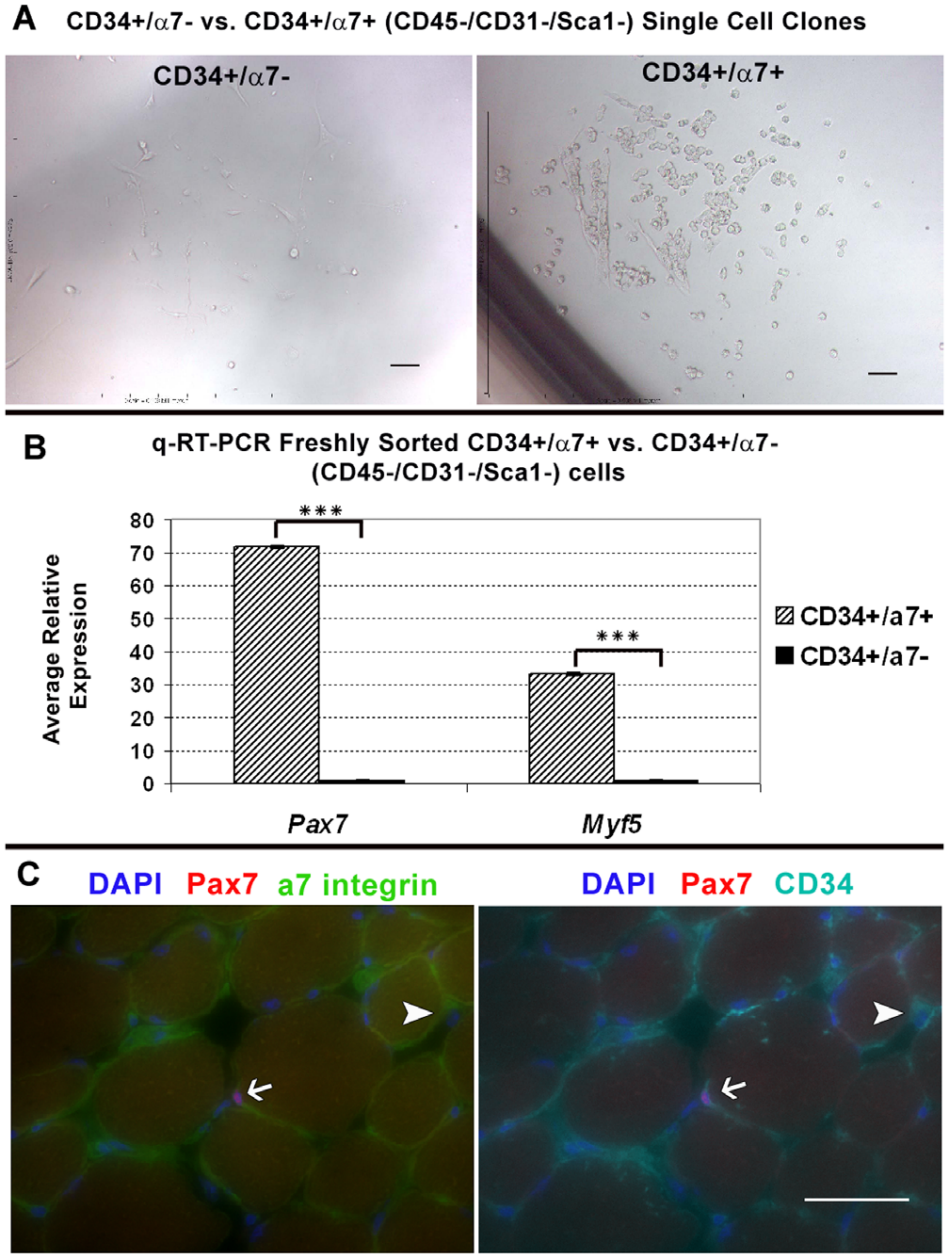
CD34+/α7 integrin- cells are not myogenic nor anatomically defined satellite cells. (A) Colonies derived from FACS-mediated single cell deposition of CD34+/α7− and CD34+/α7+ (CD45−/CD31−/Sca1−) cells (n = 3). Only α7+ sorted cells gave rise to colonies of small round myogenic cells. (B) q-RT-PCR of freshly sorted cells (n = 2) indicates only α7+ cells express *Pax7* and *Myf5.* *** denotes P≤0.00005 calculated by Student's *t*-test. Error bars represent ±SEM. (C) Immunostained TA cryosections reveal Pax7+ satellite cells are CD34+/α7+ (arrow) while CD34+/α7− cells sighted within interstitium were Pax7- (arrow head). Negative controls for all stainings are depicted in Figure S4. Scale bars = 50 µm.

To improve satellite cell purification we incorporated α7 and investigated the myogenic potential within the CD45−/CD31−/Sca1− population based on the presence or absence of α7 and CD34 antigens. By FACS-mediated single cell deposition only CD34+/α7+ (CD45−/CD31−/Sca1−) sorted cells, which were small and round, gave rise to myogenic colonies (n = 3). In contrast, CD34+/α7− (CD45−/CD31−/Sca1−) cells appeared larger and more spread-out and did not form myotubes (n = 3)(Figure 1A). Quantitative reverse-transcription PCR (q-RT-PCR) of freshly sorted cells (n = 2) revealed that between these two populations, only CD34+/α7+ cells express the definitive satellite cells genes *Pax7* and *Myf5* (Figure 1B). Cryosections from uninjured tibialis anterior (TA) muscle, immunostainied for Pax7, α7 and CD34, revealed the presence of both populations. However, only CD34+/α7+ cells were Pax7+ and within the satellite cell position. CD34+/α7− cells were Pax7− and located in the interstitium (Figure 1C). Altogether, our results indicate CD34+/α7+ cells are present in the satellite cell compartment while CD34+/α7− cells are not myogenic in culture nor satellite cells by anatomical definition.

By FACS-analysis we determined that α7− cells constitute 11.8% (±1.2% SEM) of the CD45−/CD31−/Sca1−/CD34+ population (n = 5); note with the exception of transplant studies and reporter mice, all experiments characterizing CD34+/− cells were completed with 2 month old C57BL/6 male mice. We believe sorted CD34+/α7− cells, which correspond with CD34+/α7−/Pax7− cells observed by immunostaining, were the source of non-myogenic CD45−/CD31−/Sca1−/CD34+ cells. Collectively, our results are consistent with previous reports that CD34 is present on the majority of skeletal muscle satellite cells. However, because CD34 cell surface expression is not exclusive to satellite cells, it is insufficient to FACS purify satellite cells unless used in conjunction with a second positive antigen such as α7.

### CD34−/α7+ (CD45−/CD31−/Sca1−) Cells Represent a Minority of Limb Muscle Pax7+ Satellite Cells

**Figure 2:**
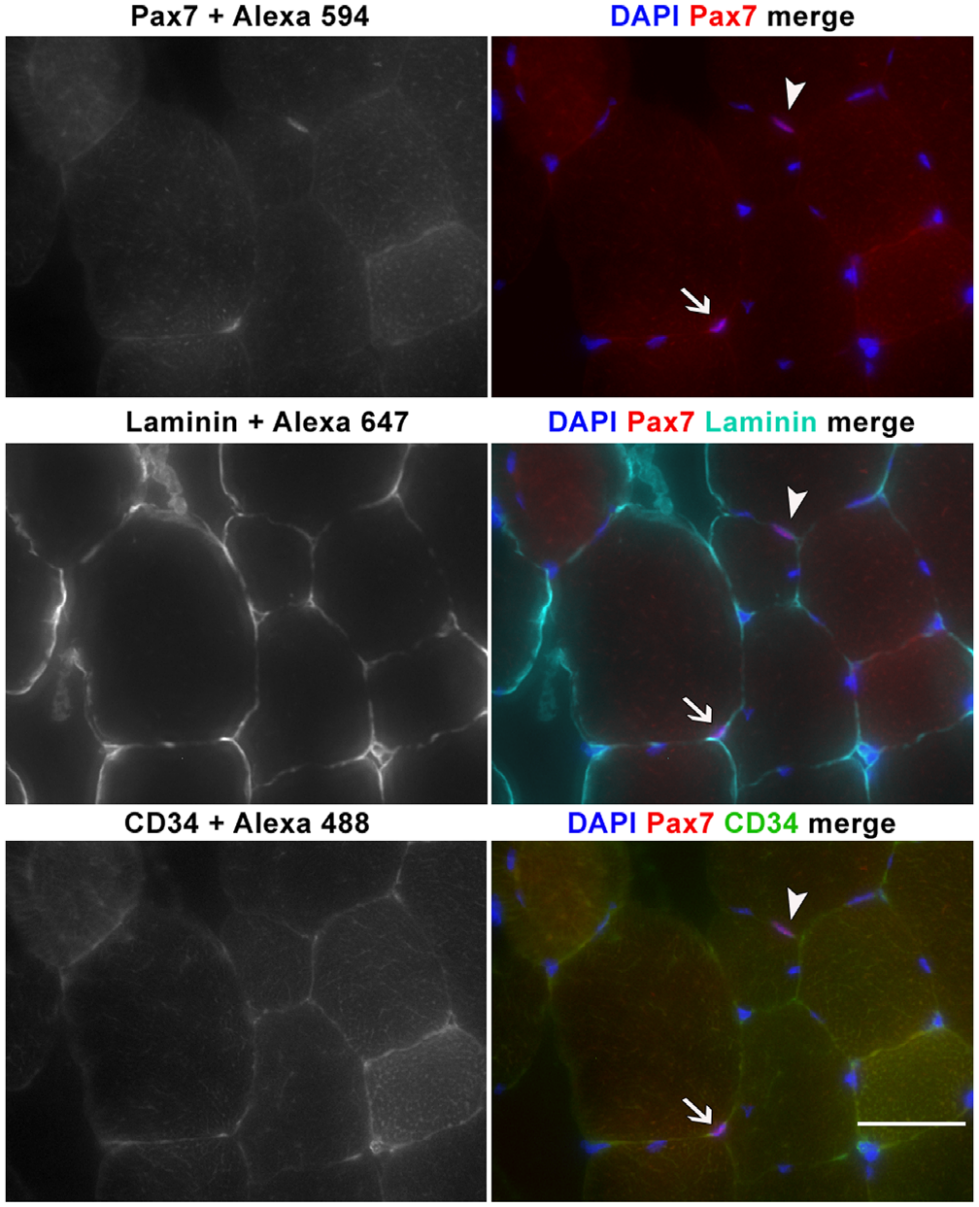
CD34+ and CD34- cells are Pax7+ satellite cells of the skeletal muscle. Cryosections of tibialis anterior (TA) muscle immunostained for Pax7, laminin, and CD34 show the existence of CD34− satellite cells. Both CD34−/Pax7+ (arrow head) and CD34+/Pax7+ (arrow) cells were sighted in the satellite position under the basal lamina. Scale bar = 50 µm.

When purifying α7+ (CD45−/CD31−/Sca1−) cells, we observed CD34+ majority and CD34− minority populations, both of which produced myogenic colonies in culture. These CD34+/− populations sorted as CD45−/CD31−/Sca1−/α7+ shall be abbreviated CD34+ and CD34− for the remainder of this manuscript. FACS-analysis indicates CD34− cells make up ∼17.6% (±2.2% SEM) of the α7+ (CD45−/CD31−/Sca1−) population in pooled muscle preparations from individual mice (n = 5). Further antibody staining revealed the presence of CD34−/Pax7+ cells in the satellite cell position underneath the basal lamina; confirming CD34− cells are an integral part of the satellite cell niche *in vivo* and not a mere product of cell isolation procedures (Figure 2). Satellite cells constitute only 2–5% of the nuclei in adult muscles and their abundance varies between muscle type and age [24], [25]. Therefore, direct quantification from cross sections would have required that many samples and cross sections be analyzed for accurate numbers of CD34+ and CD34−, Pax7+ satellite cells. Thus in order to quantify the abundance of these two satellite cell populations, we performed flow cytometry analysis of pooled muscles as well as individual muscles.

**Figure 3:**
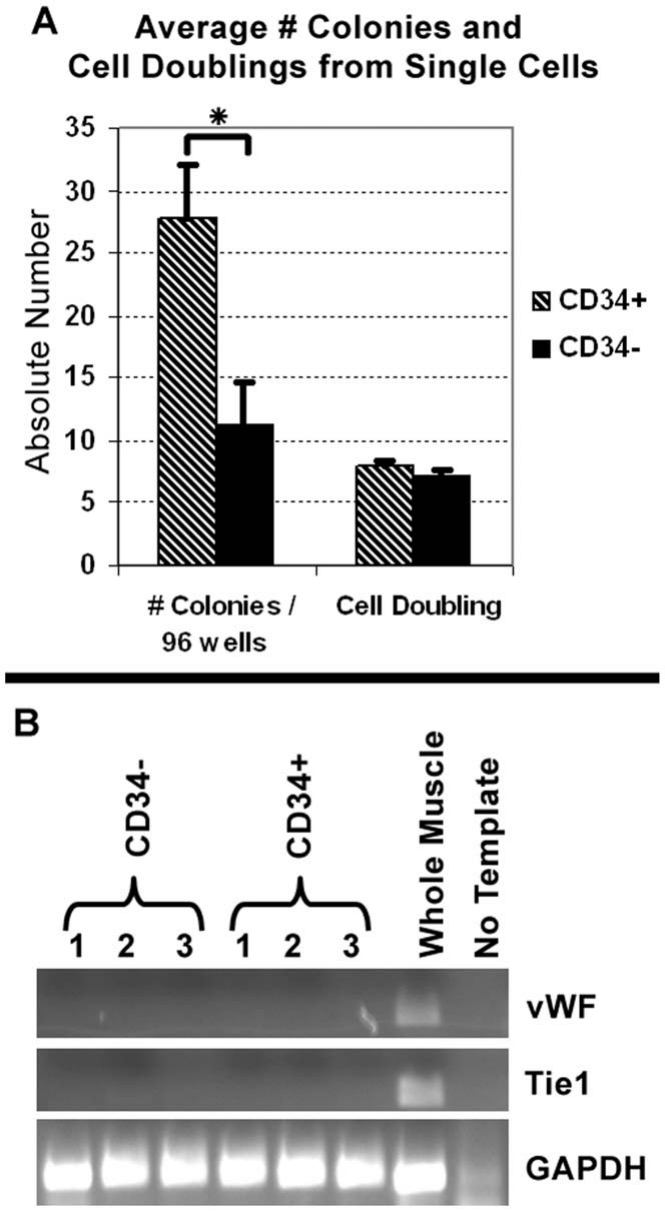
CD34+/− sorted cells represent clonal non-endothelial myogenic populations. (A) FACS-mediated single cell deposition indicates both populations had similar proliferation rates, although CD34+ cells gave rise to more colonies. Y-axis denotes the average number of colonies per 96 well plate or cell doublings. Although CD34+ single cells gave rise to more colonies, both populations were equally myogenic in culture (B) RT-PCR of freshly sorted cells for endothelial cell expressed genes *von Willenbrand Factor* (*vWF*) and *Tie1* confirms no contamination from CD34+/α7+ capillaries. Both A and B are from 2 month old, n = 3 C57BL/6 mice. Student's *t*-test, * P = 0.05. Error bars represent ±SEM.

Analysis of myogenic colonies generated by FACS-mediated single cell deposition of CD34+/− satellite cells (1 cell per well, 96 wells per cell type from each mouse, n = 3) revealed that both populations contributed myogenic clones with similar growth pattern based on the number of nuclei counted from each colony (Figure 3A, cell doubling). However, clonability was higher in the CD34+ versus the CD34− population (Figure 3A, #colonies/per 96 wells). The difference observed between the number of wells with colonies, suggests the CD34+ population exhibits greater cloning efficiency. Alternatively, our culture conditions may not be optimal for seeding CD34− sorted cells.

As noted above, all Pax7+ cells were α7+ (Figure 1C). However, not all α7+ cells were Pax7+. The microvascular bed, especially vascular smooth muscle cells stained positive for α7 (data not shown)[26], [27]. However, in the event that any endothelial cells may be α7+, such putative endothelial cells are CD31+ and are excluded from the CD45−/CD31−/Sca1−/α7+ population. Indeed, RT-PCR of freshly sorted cells (n = 3) for *von Willebrand Factor* and *Tie1,* genes expressed by skeletal muscle endothelium, confirms the absence of endothelial cells within both sorted cell populations (Figure 3B) [20]. Additionally vascular smooth muscle cells within skeletal muscles immunostained negative for CD34 and thus were presumed to be excluded from the sorted CD34+ population (not shown)[28]. Although such vascular smooth muscle cells could potentially be present in the CD34− satellite cell population, our sort approach should eliminate the presence of such cells from the final population. As previously published, satellite cells have also been defined as small non-granulated cells based on FACS-analysis [13]. Likewise, in our FACS isolation of satellite cells, we gate on smaller events by forward scatter (FSC)-Area vs. FSC-Height to remove larger α7+ cells that may originate from vascular smooth muscle (Figure S1).

**Figure 4:**
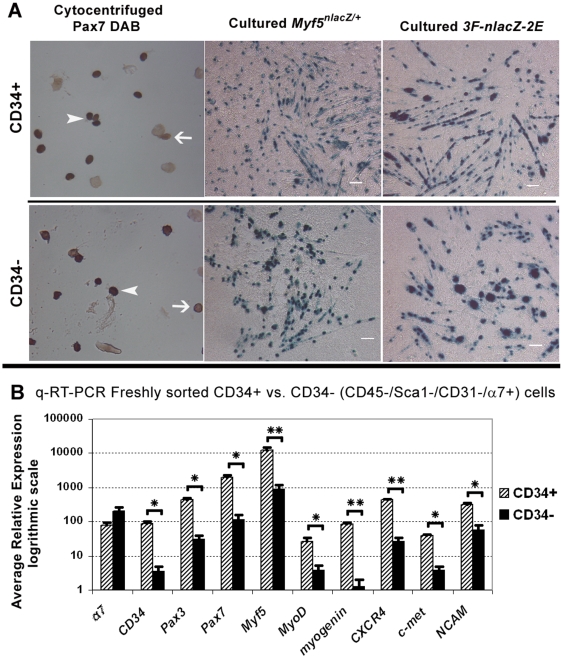
CD34+/− sorted populations contain a similar number of satellite cells that vary in gene expression. (A) The majority of freshly sorted cytocentrifuged CD34+/− cells stained positive for Pax7 DAB; 89% (±1.7% SEM) of CD34+ and 70% (±2.6% SEM) of CD34− cells were positive (n = 3, 2 month old C57BL/6). Although this difference was statistically significant (Students *t*-test, P≤0.05), the majority of CD34+ and CD34− cells were Pax7+ indicating a high purity of satellite cells within both populations. Arrowheads indicate Pax7+ cells while line arrows point to Pax7- cells. Cultured CD34+/− cells from *Myf5^nlacZ/+^* and *3F-nlacZ-2F* mice gave rise to β-galactosidase+ myogenic colonies as indicated by X-gal staining. The presence of *Myf5^nlacZ/+^* and *3F-nlacZ-2F* reporters in culture suggests both populations have similar myogenic potential. 60× scale bar for cytocentrifuged photos was unavailable. Scale bars for cultured cells = 50 µm. (B) q-RT-PCR of freshly sorted cells confirms *α7* expression in both populations and low *CD34* expression in CD34− cells (n = 3, 2 month old C57BL/6). Although higher in CD34+ cells, both populations expressed satellite cell markers *Pax3/7,* and myogenic regulatory factors *Myf5, MyoD,* and m*yogenin*. Expression of satellite cell associated genes *CXCR4*, *c-met*, and *NCAM* was also higher in CD34+ cells. Student's *t*-test calculated * P≤0.05 and ** P≤0.005. Error bars represent ±SEM.

To assess satellite cell purity within each population, we cytocentrifuged freshly sorted CD34+/− cells (n = 3 mice) and immunostained with anti-Pax7. From both sorted populations we obtained a high purity of myogenic cells as indicated by the presence of nuclear Pax7 staining (Figure 4A, left panels). On average, 89% (±1.7% SEM) of CD34+ and 70% (±2.6% SEM) of CD34− cells stained positive for Pax7. Although the majority of cytocentrifuged cells from both populations stained positive, the difference in the frequency of Pax7+ cell was significant (Student's *t*-test, P = 0.04) indicating there is some contribution of Pax7- cells within the CD34− population.

We also cytocentrifuged CD34+ and CD34− cells sorted from adult *3F-nlacZ-2F* reporter mice to assess the possible presence of differentiated progeny of satellite cells in the CD34+/− populations. In this transgenic mouse, muscle fibers and cultured differentiated myoblasts and myotubes express nuclear localized *LacZ* [5], [15], [29]. Nuclei positive for β-galactosidase (β-gal) were identified by X-gal staining (data not shown). We observed that CD34− cells were completely negative, while approximately 0.01% of CD34+ cells stained positive for β-gal activity with X-gal. With the exception of the few CD34+/β−gal+ cells observed, lack of *3F-nlacZ-2F* reporter activity indicated the vast majority of cells in the CD34+/− sorted populations were not differentiated myoblasts (the phase at which this transgene is activated) [5], [29]. In contrast, we did identify β-gal+ cells from cytocentrifuged *3F-nlacZ-2F* unsorted cells stained with X-gal as a positive control (not shown).

To assess the *in vitro* myogenic potential of these two populations, we cultured CD34+/− sorted cells from *3F-nlacZ-2F* (n = 2, pooled) and *Myf5^nlacZ/+^* mice (n = 3). Cultured CD34+/− cells sorted from *Myf5^nlacZ/+^* and *3F-nlacZ-2F* mice produced progeny that looked identical in culture (Figure 4A, middle and right panels). From *Myf5^nlacZ/+^* mice, both populations formed myogenic colonies with numerous β-gal+ myonuclei and myoblasts. Myonuclei from *Myf5^nlacZ/+^* mice stained positive with X-gal due to the presence of residual β-gal expressed also in differentiating cells [15]. In contrast, *3F-nlacZ-2F* reporter activity was predominantly localized in myonuclei.

To further compare the CD34+/− populations, we surveyed the expression of genes known to be transcribed by satellite cells (Figure 4B). Due to possible gene regulation changes in culture that may not necessarily represent *in vivo* expression, we chose to analyze only freshly sorted cells by q-RT-PCR. Initially we checked *CD34* and *α* *7* expression to validate our FACS-analysis (Figure 4B). In accordance with antigen levels, CD34− cells express very low levels of *CD34* mRNA, whereas CD34+ cells transcribe much higher levels (25 times greater than CD34− cells). The expression of *α7* was similar in both CD34+/− populations and not statistically significant (Student's *t*-test, P>0.05). Next, we compared the two populations for the expression of a panel of genes that are associated with different phases of myogenesis (Figure 4B). *Pax3*, *Pax7,* and *Myf5* are expressed by quiescent and proliferating satellite cells [4], [12], [30], [31]. *MyoD* expression is initiated upon satellite cell activation and sustained in proliferating and differentiating progeny, while *myogenin* expression is associated specifically with myogenic differentiation [11], [32]. Interestingly, expression levels of *Pax3/7* genes and muscle regulatory factors (MRF's) *Myf5*, *MyoD*, and *myogenin* were significantly higher in CD34+ vs. CD34− cells (Student's *t*-test, P≤0.05). The detection of *myogenin* suggests the presence of some differentiating myogenic cells, which may account for the presence of a minute number of cytocentrifuged CD34+ cells that stained positive with X-gal for the differentiation-linked 3*F-nlacZ-2F* reporter as detailed above. Our detection of *myogenin* and *MyoD* transcripts, in addition to *Myf5*, in preparation of freshly isolated satellite cells is in agreement with previous studies describing gene expression in freshly isolated satellite cells [33], [34]. Expression of satellite cell associated genes *CXCR4, c-met* and *NCAM* was also significantly higher in CD34+ cells (Student's *t*-test, P≤0.05). Collectively, this expression comparison suggests that CD34+ cells transcribe higher levels of satellite cell characteristic genes than CD34− cells. Alternatively, the CD34− population may contain a residual population of non-myogenic cells that contributes to the apparent reduced expression of these genes.

### The CD34+ and CD34− Populations are Consistently Maintained Across Different Muscles

**Figure 5:**
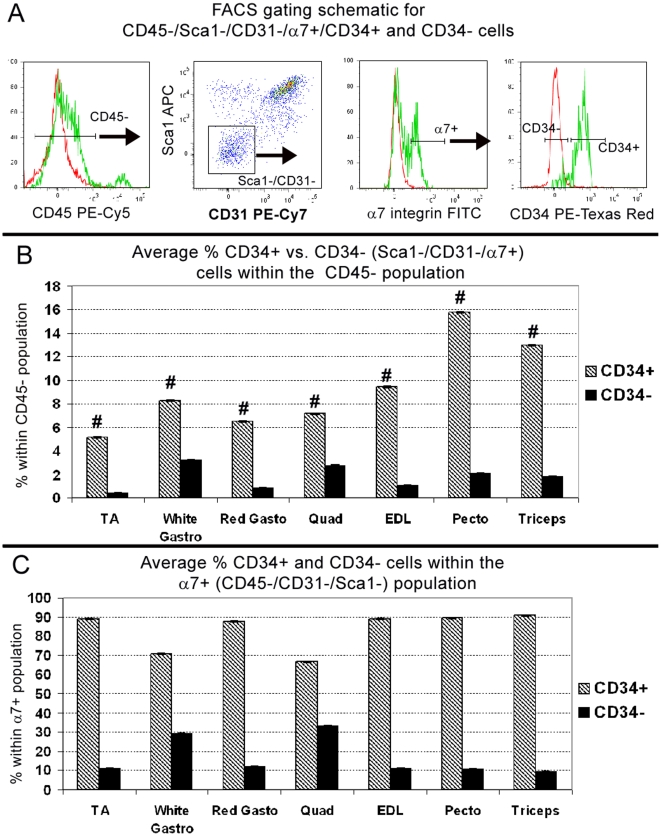
The distribution of CD34+/− cells is maintained among individual muscles. (A) Gating schematic for sorting and analyzing CD34+/− populations. Following size selection, hematopoietic cells are removed by selecting only CD45− cells. Endothelial and other non-satellite cells are then removed by gating on CD31−/Sca1− cells. Finally within the α7+ (CD45−/CD31−/Sca1−) population, CD34+/− cells are selected. Red peaks represent unstained controls, green peaks are experimental stained sample. (B) The average percentage of each population per muscle group relative to all CD45−, non-hematopoietic cells. By single factor ANOVA, differences between muscles was statistically significant for CD34+ (# P≤0.05) but not CD34− (P = 0.076). (C) The average percentage of CD34+ and CD34− cells within the α7+ (CD45−/CD31−/Sca1−) population. The ratio of CD34+ over CD34− cells was not statistically significant by single factor ANOVA. A–C are from n = 5, 2 month old C57BL/6 mice. Error bars represent ±SEM.

In the aforementioned experiments, we isolated CD34+/− cells from pooled hindlimb, pectoralis and triceps muscles. In contrast, the study by Beauchamp et al. (2000) quantified the percentage of CD34+ satellite cells only on EDL single fiber isolations. Thus, to address the possibility that CD34− satellite cells reside only in specific skeletal muscles, we FACS-analyzed individual muscles (n = 5) using same selection strategy used for pooled muscle isolations (Figure 5A). Because the number of hematopoietic cells can vary between mice depending on circulatory responses, such as mild inflammation, we restricted our analysis to the CD45− fraction. For each muscle, Figure 5B represents the average percentage of CD34+/− cells compared to the total number of non-hematopoietic mononuclear cells. Figure 5C represents the average percentage within the α7+ (CD45−/CD31−/Sca1−) population in order to determine if CD34+ cells consistently represent the majority.

Results from individual muscle analyses indicate that the average percentage of CD34+ cells compared to the CD45− fraction is consistent between hindlimb muscles, whereas pectoralis and triceps contain nearly twice the amount of CD34+ cells. Such variation in the number of CD34+ cells may be attributed to individual muscle demands for myogenic cells and/or myofiber types. Consequently, the percentage of CD34+ cells was statistically different (single factor ANOVA, p≤0.05) between muscle groups, whereas the percentage of CD34− cells was not (P = 0.075), indicating this population is consistently present in all muscle groups analyzed. Despite this variation, CD34+ cells were consistently the majority within the α7+ (CD45−/CD31−/Sca1−) population. The ratio of CD34+ over CD34− cells was not statistically significant (single factor ANOVA, p>0.05), suggesting the distribution of these two populations is maintained between muscle groups.

### Following Acute Injury, CD34− Cells Represent the Majority of Activated α7+ (CD45−/CD31−/Sca1−) Myogenic Cells

**Figure 6:**
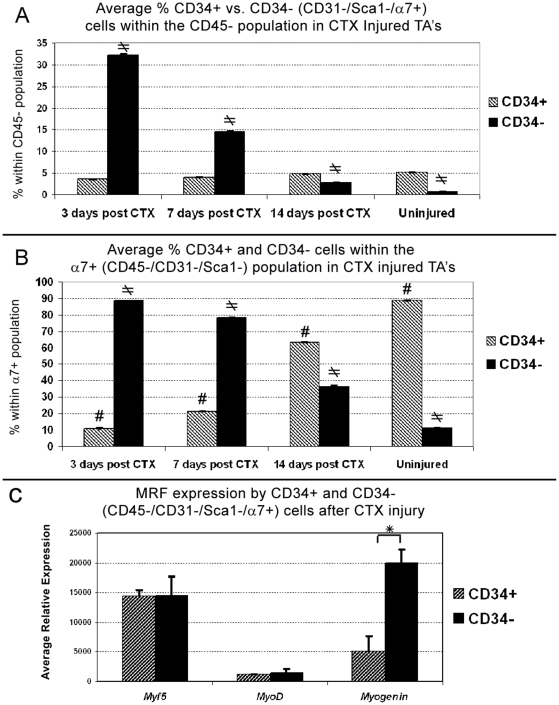
Following acute injury, CD34− cells are the more responsive myogenic majority. (A) Relative to all CD45− cells, the average percentage of CD34− cells in Cardiotoxin (CTX) injected TA's (n = 5 per time point) significantly increased 3 days post injury and declines to near uninjured levels by day 14 (single factor ANOVA, ≠P = ≤0.05). In contrast, the percentage of CD34+ cells remained relatively constant during course of regeneration and was not statistically significant. (B) 3 days post injury the average percentage of CD34− within the α7+ (CD45/CD31−/Sca1−) population drastically increases. This proportion reverts back to near uninjured levels by day 14 and by single factor ANOVA was statistically significant for both CD34+ (# P≤0.05) and CD34− (≠P≤0.05) populations. (C) q-RT-PCR analysis of cells sorted 3 days post injury from CTX treated limb muscles (n = 3), indicates the average relative expression of all three MRF's increased in both populations as compared to uninjured muscles depicted in Figure 4B. Comparison of MRF's levels in injury reveals both populations express equal levels of key myogenic genes *Myf5* and *MyoD,* while CD34− cells express significantly elevated levels of the differentiation factor *myogenin*. Student's *t*-test, * P≤0.05. Error bars represent ±SEM. A–C are from 2 month old C57BL/6 mice.

To elucidate the role of CD34− skeletal muscle cells of the skeletal muscle we examined their response to acute injury using the cardiotoxin (CTX) model. Such injury is characterized by a massive myogenic response leading to almost full muscle regeneration by week two following initial tissue destruction [35], [36]. We injected CTX in left TA's and FACS-analyzed each damaged muscle individually at 3, 7, and 14 days post injury (n = 5 per time point)(Figure 6A&B). Just as the previous analysis of individual muscles, we compared here the percentage of CD34+/− cells within the CD45− fraction (Figure 6A) and the relative percentage within all α7+ (CD45−/CD31−/Sca1−) cells (Figure 6B). For this experiment we predicted that the more differentiated population will increase early after injury and continuously decrease from day 7 to14 as the muscle reaches full regeneration. In addition, if there is a canonical relationship between CD34+/− cells, we have anticipated an inverse trend where one population decreases while the other increases during the course of regeneration.

As expected, 3 days post injury there was a massive increase in the number of α7+ (CD45−/CD31−/Sca1−) cells which by histology were not vascular smooth muscle cells as indicated by α-smooth muscle actin (αSMA) staining (Figure S2). Surprisingly after CTX injury, the CD34- cells became the majority and declined to near uninjured levels by day 14. In contrast, the average percentage of CD34+ cells remained between 3–5% within the CD45− fraction (Figure 6A) despite becoming the minority within α7+ (CD45−/CD31−/Sca1−) population (Figure 6B). The fluctuation of CD34− cells between time points within CD45− fraction and α7+ population was statistically significant (single factor ANOVA, p≤0.05). However, the percentage of CD34+ cells within the CD45− fraction remained relatively constant (p = 0.076).

Since CD34+ cells became the minority within the α7+ (CD45−/CD31−/Sca1−) population early in injury but the overall percentage of these cells did not increase, we postulated this population remained quiescent while CD34− cells were activated. Thus, we compared *Myf5*, *MyoD*, and *myogenin* expression by q-RT-PCR in freshly sorted cells from damaged limb muscles at 3 days post CTX injury (Figure 6C). Cells were sorted from pooled CTX injected muscles (single TA, quadriceps and gastrocnemius) from individual mice (n = 3). Without injury both CD34+/− cells expressed relative high levels of *Myf5* but low levels of *MyoD* and *myogenin* mRNA, indicating both populations are committed to the myogenic pathway, but remain quiescent in uninjured muscle (Figure 4B). In contrast, following injury the average relative expression of all three MRF's increased in both populations (Figure 6C). *Myf5* and *MyoD* expression was similar between both populations whereas *myogenin* was 3.9 times greater and statistically significant (Student's *t-*test, p≤0.05) in CD34− cells. In accordance with MRF expression during myogenesis, upregulation of *Myf5* and *MyoD* following injury indicates both CD34+/− cells are activated while higher *myogenin* expression suggests that more differentiated cells reside within the CD34− population [11].

**Figure 7:**
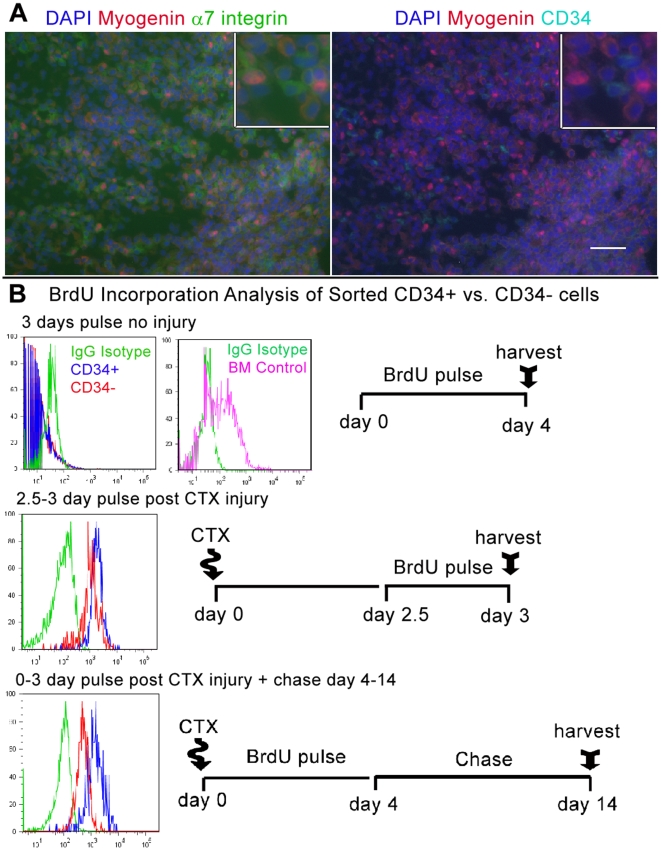
Although CD34− cells were more differentiated, both CD34+/− populations proliferated in response to injury. (A) Immunostaining of damaged TA, 3 days post injury, reveals that myogenin+ cells within the sight of injury are α7+/CD34−. Insets show co-localization of DAPI with nuclear myogenin and membranous α7 but not CD34 staining. Insets were produced from the original sized merged photographs. Scale bar = 50 µm. (B) FACS-analysis of BrdU incorporation in sorted CD34+/− cells after 3 days pulse, indicates relative to bone marrow (BM) cells (top right histogram), without injury both populations are quiescent (top histogram). In mice pulsed for 12 hours between 2.5–3 days post CTX injection, both CD34+/− cells show nearly equal levels of BrdU incorporation (middle histogram). Mice pulsed for the first 3 days following injury and then chased to day 14 reveal that CD34− cells retain less BrdU whereas CD34+ cells maintained a similar levels to the day 2.5–3 pulse (bottom histogram). Green peaks represent IgG Isotype controls, Violet BM mononuclear cells, Blue CD34+ cells, and Red CD34− cells. Schematics next to histograms portray timelines for each BrdU experiment. Each experimental pulse was conducted with n = 3, 2 month old C57BL/6 mice.

The significance of *myogenin* expression was confirmed by immunostaining as the majority of myogenin+ cells observed within damaged areas 3 days post CTX injury, were α7+/CD34− (Figure 7A). Taking into account myogenin histological and FACS data we concluded CD34− cells were directly responding to injury. In contrast, significant upregulation of *MyoD* but not *myogenin* by CD34+ cells suggested this population was activated but not differentiating. However, the role of CD34+ cells following injury was unclear as their overall numbers did not increase while the majority of α7+ cells observed within damaged areas were CD34−.

### Although Both CD34+/− Populations Proliferate in Response to Injury, CD34+ Cells Retain More BrdU

Because the percentage of CD34+ cells did not increase with CTX damage, it was possible that there was a general increase in all cell types or that CD34+ cells did not proliferate in response to injury. To explore this alternative, we compared BrdU incorporation in order to quantify proliferation between CD34+/− cells and asses if the absence of CD34 simply marks mitotically active myogenic cells. In order to test this hypothesis we analyzed BrdU incorporation without injury, 2.5–3 days post injury within the peak of proliferation, and after regeneration 14 days post injury (n = 3 for each timepoint analyzed)(Figure 7B)[35]. Bone marrow cells isolated from the same animals as a positive control had a high degree of BrdU incorporation following 3 days pulse. In contrast, BrdU was not detected in CD34+ or CD34− cells from uninjured limb. However, upon injury, BrdU was incorporated into the CD34+/− populations. When isolated from mice pulsed for 12 hours between day 2.5–3 post CTX injury, both CD34+/− populations had incorporated equally high levels of BrdU.

Interestingly, in cells isolated from mice pulsed for the first 3 days following injury and then chased to day 14, BrdU incorporation declined in CD34− cells but remained the same for CD34+ cells. Consequently, the amount BrdU retention by day 14 was roughly 10 fold greater for CD34+ vs. CD34− cells as represented by the log distance from the mean of each fluorescence peak. Given that the level of BrdU uptake at day 2.5–3 was similar for both populations, we estimate that the CD34− population divided >3 cell doublings during the day 4–14 chase.

Without injury, the lack of detectable BrdU incorporation correlates with q-RT-PCR data to indicate both populations are quiescent. Following injury both populations have equal levels of BrdU incorporation indicating a similar degree of cell division occurred within the peak of proliferation. Beyond 3 days after injury, CD34+ cells retained a similar level of BrdU indicating they ceased dividing, while CD34− continued to proliferate as their incorporation decreased during the chase. Interestingly, although CD34+ cells did incorporate BrdU following injury, their percentage did not increase but remained relatively constant. Therefore we postulated CD34+ cells give rise to more committed cells while tightly maintaining the parent population during the course of regeneration. All together, FACS, histological, and BrdU results indicate CD34+ cells divided early in response to injury and perhaps gave rise to CD34− cells, which further proliferated during the course of regeneration.

### CD34+/− Switching is Dependent on Muscle Injury

**Figure 8:**
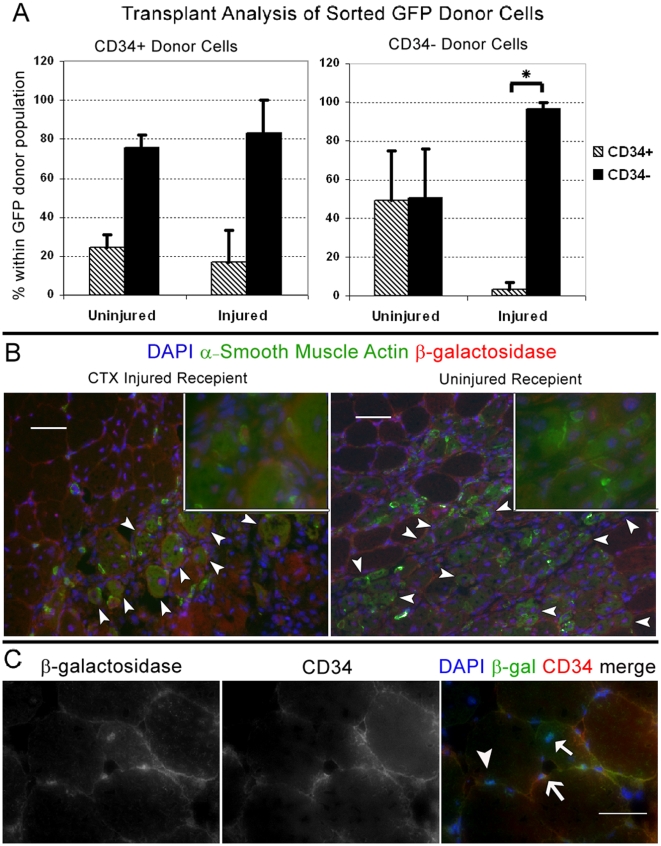
Following injury repair CD34− cells can revert to CD34+. (A) CD34+/− cells sorted from injured (3 days prior) limb muscles of chicken actin promoter driven EGFP reporter mice, were transplanted into uninjured and injured (1 day prior) quadriceps of C57BL/6 recepients. Quadriceps were FACS-analyzed 3 days post transplant. Values in the Y-axis correspond to the percentage of GFP CD34+/− cells detected from each respective donor cell type. The X-axis indicates the state of each recipient muscle prior to transplant (n = 3 per condition). Irrespective of the recipient state, the majority of CD34+ donor cell became or gave rise to CD34− after transplant. Although not statistically significant, this switch slightly increased in CTX injured recipients. In contrast a large proportion of CD34− cells became or gave rise to CD34+ when transplanted into uninjured muscle but significantly remained CD34− in injured muscle. Student's *t*-test calculated * P≤0.05. Error bars represent ±SEM. (B) Sorted CD34− from CTX injured *Myf5^nlacZ/+^* mice were transplanted into uninjured (n = 3) and injured (n = 3) quadriceps muscles. In addition to a mock injection, α7- (CD45/CD31−/Sca1−) sorted cell were transplanted into uninjured quadriceps as negative controls (Figures S3 and S4). Recipient muscles were harvested 7 days after transplant and stained for α-smooth muscle actin (αSMA), β-galactosidase (β-gal) and CD34. Cells engrafted under both conditions as indicated by β-gal+ central nuclei in αSMA+ fibers (arrowheads). Insets show co-localization of nuclear β-gal with DAPI and cytoplasmic α-SMA staining. Insets were produced from 40× magnification photographs taken within the same field. (C) Engraftment of CD34- cells into uninjured muscle reveals conversion of CD34− cells to CD34+ cells. Interestingly CD34+ (line arrow) and CD34− (arrowhead), β-gal+ cells were observed near β-gal+ myonuclei (solid arrow) indicating donor CD34− cells can form new myofibers, remain CD34− or gain CD34 expression. Scale bars = 50 µm.

To test the possibility that activated CD34+ cells are becoming or giving rise to CD34− cells, we sorted both populations from injured chicken actin promoter driven EGFP transgenic mice and transplanted into injured and uninjured quadriceps of C57BL/6 mice**.** Recipient muscles were then analyzed by FACS to examine if GFP donor cells gained or lost CD34 when transplanted into each respective environment. Injured recipient quadriceps were injected with CTX one day prior to cell transplants. All recipient muscles were analyzed 3 days post transplant in order to detect donor cells prior to fusion (n = 3 for each condition). CD34 analysis of transplanted GFP cells indicated a distinct pattern of switching dependent on the state of muscle injury. As predicted, the majority of injury activated CD34+ donor cells gave rise to, or directly became CD34 negative. The percentage of CD34− cells generated from CD34+ donors was slightly more in injured recipient muscles, although this difference was not significant (Student's *t-*test, p>0.05)(Figure 8A, left graph). Conversely, the majority of CD34− cells transplanted into injured muscle remained CD34− (Figure 8A, right graph). To our surprise a large portion of CD34− cells transplanted into uninjured muscle became and/or gave rise to CD34+ cells. In contrast to the CD34 expressing phenotype acquired from negative donors following transplant into uninjured muscles, the percentage of CD34− cells detected in transplanted injured muscles was very consistent and statistically significant (Student's *t-*test, P≤0.05)(Figure 8A, right graph). These results indicated that the presence or absence of CD34 on satellite cells is conditionally dependent on muscle environmental demands and not necessarily hierarchal as initially hypothesized.

**Figure 9:**
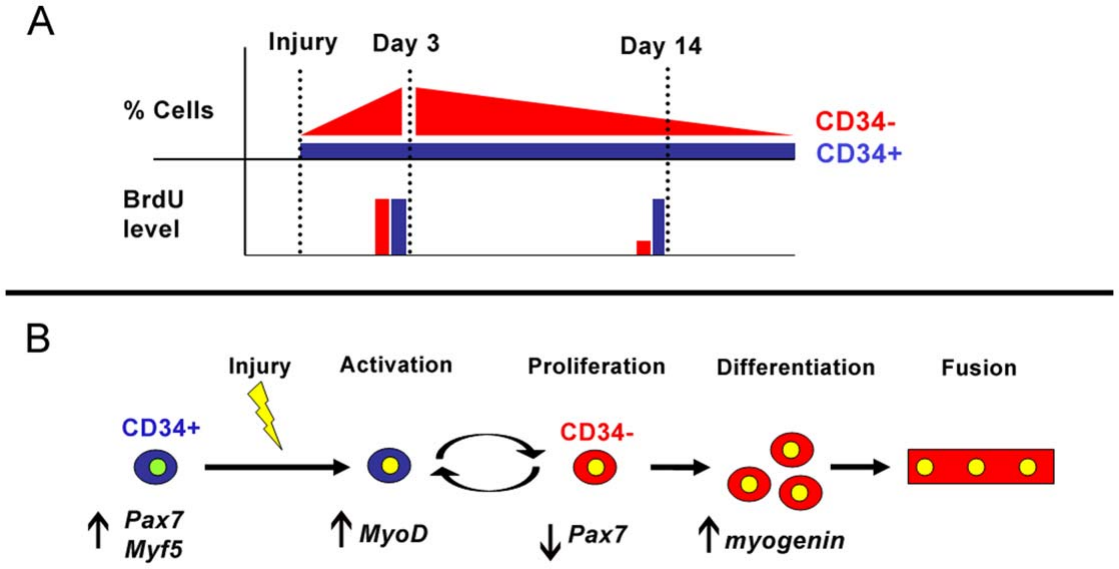
Summary of significant results and proposed model of CD34 switching by satellite cells following injury. (A) Following acute injury the proportion of CD34− cells drastically increases while the CD34+ populations remains to near uninjured levels. Despite the increase of only CD34− cells, BrdU analysis indicates both populations proliferate shortly after injury (day 3) while CD34+ cells retain BrdU following regeneration (day 14). Such trends indicate that CD34+ population represents the pool of less advanced satellite cells that can generate more differentiated CD34− cells while maintaining their numbers during injury repair. In turn, CD34− cells continue proliferating beyond day 3, indicating this population supplies the differentiated progeny required for complete muscle regeneration. (B) Although more differentiated cells reside within the CD34− population following injury, the absence of CD34 does not terminally mark differentiation as CD34− cells can revert back to the CD34+ pool of less advanced satellite cells. Thus activated myogenic cells can switch CD34 expression in response to injury, suggesting that CD34 may play a role in maintaining a reserve pool of satellite cell in order to prevent depletion during muscle regeneration.

Because we only studied a short timeframe after injury, it was unclear if later during the course of regeneration, CD34− cells ever transitioned back to CD34+. To examine the possibility of conversion following the peak of proliferation, we sorted and transplanted CD34− cells isolated from muscles of *Myf5^nlacZ/+^* mice 3 days post CTX injury directly into uninjured (n = 3) and injured (n = 3) quadriceps injected the previous day with CTX (C57BL/6 recipients). In order to detect the *Myf5^nlacZ/+^* transgene in donor cells that may have recently fused or formed immature myofibers, we harvested recipient muscles 7 days post transplant. Cyrosections were initially stained with X-gal to identify and validate the presence of *Myf5^nlacZ/+^* donor cells. Once β-gal+ cells were identified (Figure S3), adjacent cryosections were stained with antibodies against β-gal, CD34, and αSMA as a marker of newly regenerated myofibers [37], [38], [39]. Results indicate that activated CD34− donor cells were myogenic as in both injured and uninjured recipient muscles they formed new muscle fibers identified as α-SMA+ with β-gal+ centrally located nuclei (Figure 8B). In addition, we observed mononuclear β-gal+/CD34+ cells indicating some CD34− cells revert back to a reserve state following injury repair (Figure 8C). Altogether, the analysis of the FACS population dynamics during regeneration and BrdU pulse-chase studies, suggest that the CD34− population is the major myogenic population responsive of regeneration after injury. In addition, the transplant studies indicate that some CD34− negative cells can localize to the satellite cell position and express CD34. We propose a mechanism for this reversible switching of CD34 in response to injury to generate enough differentiating progeny while maintaining the pool of reserve satellite cells during regeneration (Figure 9).

## Discussion

In recent years several surface receptors, including CD34, have been described to recognize satellite cells of the skeletal muscle [5]. However, no single surface receptor has proven exclusive to satellite cells, and CD34 is no exception. By utilizing FACS we set out to isolate pure populations of satellite cells using a host of antigens in order to select out contaminating cells that also express CD34. Our results reveal the population of skeletal muscle cells characterized as CD45−/CD31−/Sca1−/CD34+/α7−, lack expression of myogenic transcription factors *Pax7* and *Myf5*, do not form myotubes *in vitro*, nor conform to the satellite cell anatomically defined position under the basal lamina. Therefore, we conclude CD34 alone is inadequate for selecting pure populations of satellite cells from the skeletal muscle.

Since the initial observation by Beauchamp et al. (2000), CD34 has been widely used to identify and isolate satellite cells [9], [13], [19], [40], [41], [42]. Contrary to the multitude of studies that have examined CD34+ satellite cells, the CD34− minority remained uncharacterized. Initially, we predicted that similar to the hematopoietic system, a linear relationship existed within the satellite cell compartment of skeletal muscle, where more primitive CD34− cells give rise to more committed CD34+ cells. Contrary to our initial hypothesis, our results indicate that following acute injury CD34− cells represent the more myogenically active population. Furthermore, the absence or presence of CD34 is reversible depending on the state of injury.

Currently, an inducible CD34 Cre-Lox reporter model is not available to directly distinguish between downregulation of CD34 or asymmetric division as possible means for CD34 conversion. However, BrdU analysis, myogenin expression/staining and transplant data indicate CD34− cells directly respond and contribute to muscle regeneration following acute injury. Remarkably, despite similar BrdU incorporation at the peak of proliferation between day 2.5–3 post injury, the percentage of CD34+ cells did not change over the course regeneration. Because the absence of CD34 marks a state of activation which seems reversible, the steady percentage of CD34+ cells throughout injury suggests a proportion of myogenic cells are programmed and/or stimulated to maintain a reserve pool of satellite cells during the course of regeneration. This reversible switch from activation to dormancy has also been observed with hematopoietic stem cells as they maintain the stem cell pool following bone marrow injury [43].

Despite their response following acute injury, CD34− cells were quiescent in uninjured muscle. This leaves into question the role of this population during normal muscle homeostasis. Although both CD34+/− populations were equally myogenic in culture, freshly sorted CD34− cells had lower expression of myogenic-related genes such as *Pax7*, *Pax3, Myf5, MyoD, myogenin, CXCR4*, and *c-met*. Furthermore, we and other groups have observed that within 24 hours of being seeded satellite cells lose CD34 in culture, suggesting CD34 is downregulated upon activation [5]. Therefore, CD34− cells appear to represent more “primed” satellite cells poised for injury response that can advance further in the myogenic pathway or upregulate CD34 and revert back to a reserve state depending on the muscle microenvironment. Although CD34's function has yet to be elucidated, reversibility has been reported with hematopoietic stem cells, suggesting a similar role or pathway of CD34 regulation may exists between tissue specific stem cells [44], [45]. Interestingly, despite the major myogenic potential and contribution of CD34+/− cells to muscle regeneration, a minority of Sca1+ (CD45−/CD31−/CD34+) cells sorted from injured muscle was myogenic in culture, suggesting another possible state of transition or stem cell reservoir exists within limb muscles (data not shown)[46].

Although the true function(s) of CD34 remains uncertain, it has been described that this highly glycosylated sialomucin receptor not only plays a role in signaling but is important in promoting both cellular adhesion and repulsion [44], [47]. Interestingly in one study it has been reported that CD34 functions in mast cells to prevent adhesion [48]. Conversely a separate study has reported that CD34 functions to promote lymphocyte adhesion via L-selectin in high endothelial venule cells [49]. Thus, it is possible that the adhesion role of CD34 is dependant on the presence or absence of additional surface receptors as a means to modulate binding or repulsion by various cell types. As aforementioned satellite cells downregulate CD34 in culture, suggesting this receptor functions in maintaining an undifferentiated state. Perhaps such maintenance is linked to adhesion and therefore downregulation of CD34 is required for migration and subsequent engraftment within regions of damaged muscle.

By characterizing skeletal muscle satellite cells based on the presence or absence of CD34 we have identified a key myogenic population responsible for muscle regenerating following acute injury as CD34− (α7+/CD45−/CD31−/Sca1−). In addition we show that the fraction of CD34+ cells remains steady during homeostasis and injury regeneration. Such evidence suggests, despite much heterogeneity, the satellite cell pool is highly regulated *in vivo* to maintain a reserve pool of CD34+ (α7+/CD45−/CD31−/Sca1−) cells while producing sufficient numbers of differentiated progeny for muscle regeneration.

## Materials and Methods

### Ethics Statement

This study has been reviewed and approved by the University of Washington's Institution Animal Care and Use Committee.

### Mice and Animal Care

All mice used for satellite cell characterization (histology, FACS-analysis of individual muscles and injury, single cell culture, cytocentrifugation, BrdU and expression analysis) were C57BL/6, predominantly 2 month old males. Reporter mice used for cytocentrifugation and/or culture were 1.5 month old *Myf5^nLacZ/+^* and 4 month old *3F-nlacZ-2F* [5], [50], [51], previously described in our studies [12], [29]. Transplant donor cells were derived from chicken β-actin promoter driven EGFP or *Myf5^nLacZ/+^* reporter mice ages ranging from 6–9 month old [52]. Recipients were C57BL/6, ages ranging from 2–12 months old. Unless specified otherwise, each experimental replicate (n = ) represents samples derived from individual animals that were independently processed and analyzed. Mice were housed and maintained in a modified barrier facility located at the University of Washington. All animal experiments were performed in accordance with guidelines approved by the University of Washington's Institution Animal Care and Use Committee.

### FACS

Methods for muscle processing and FACS were followed as previously described [20]. Typically from pooled preparations including both hind limb muscles, pectorals and triceps of individual 2 month old C57BL/6 males, we recover 1.5×10^6^ mononuclear cells per gram of muscle processed. Samples were initially stained with biotinylated anti-CD34 in 100 µl PBS with 0.3% BSA per 10^6^ cells for 45 minutes (min) on ice, then washed and resuspended in an antibody cocktail including conjugated streptavidin and directly conjugated anti-CD45/CD31/Sca-1/α7 for a second 45 min incubation. For single cell deposition DAPI (Sigma) was added for viability and left in solution prior to sorting in order to remove DAPI+ non-viable cells. FACS antibody data, dilutions, combinations and machine specifications are listed in Methods S1. Data was acquired at 10,000 events per sample and cells sorted with a BD Aria I and later Aria II, both using Diva software. Subsequent analysis and flow cytometry plots were generated using FlowJo v7.2.5 (TreeStar, Inc.). Average population values and standard deviations used for error bars were calculated from the analysis of individual mice. Graphs, Student's *t*-test and ANOVA statistical analyses were generated using Microsoft Excel 2003. For transplants, culture and cytocentrifugation sorted cells were collected in culture media. For reverse and quantitative PCR, sorted cells were collected in 350 µl RLT lysis buffer (Qaigen) supplemented with β-mercaptoethanol per manufacturers instructions and immediately placed on dry ice and stored at −80°C.

### Cell Culture

Clonal cultures were prepared by FACS-Aria mediated single cell deposition into 96 well trays. Non-clonal cultures cells were directly sorted into 1.7 ml tubes containing 750 µl media and seeded manually onto 24 well dishes. All tissue culture dishes were coated with 0.67% (w/v) Type-A Gelatin (Sigma) in H_2_O and allowed to dry prior to use. Culture media consisted of Ham's F10 supplemented with 15% Horse Serum and final concentration of 2 mM CaCl_2_, 100 units/ml Penicillin with 100 µg/ml Streptomycin (all from HyClone), and 20 ng/ml bFGF (R&D). Cells were maintained at 37°C, 5%O_2_, and 5%CO_2_ and media was changed after 4 days. At day 8 in culture, cells were fixed with 2% formaldehyde (diluted formalin in PBS) and stained with DAPI for counting clones or X-gal for presence of *Myf5^nlacZ/+^* or *3F-nlacZ-2F* reporter activity. After DAPI staining, photographs of each single cell derived colony was taken and used to quantify proliferation. To avoid counting errors we utilized the ImageJ v1.40 g (Wayne Rasband, NIH) cell counter plugin (Kurt De Vos, University of Sheffield) which automatically tallies events labeled by users. Subsequently every DAPI labeled nuclei including myonuclei were accounted for in the final cell count. The average cell doubling for each CD34+/− population was calculated based on the number of DAPI+ nuclei counted per colony using the following formula in Microsoft Excel; colony cell doubling = log (# nuclei counted/2).

### Stainings

All muscle cryosections were cut 8 µm, fixed with 2% formaldehyde for 5 min, washed 3× with PBS, then stained with respective antibodies. For cytocentrifugation staining, freshly sorted cells were spun onto microscope slides at 800 g RCF, fixed with 4% formaldehyde for 5 min, washed, and then stained. Stainings utilizing biotinylated antibodies were first blocked with Vector labs Streptavidin-Avidin blocking kit. Stainings using mouse primary antibodies were completed using the M.O.M staining kit (Vector labs) with some modification to manufacturer's instruction. Cryosections were incubated overnight at 4°C with the M.O.M. mouse IgG blocking reagent and streptavidin conjugated fluorophores were used in place of avidin conjugates. All antibodies were diluted in PBS containing 1% BSA (fraction IV, from Fisher) except when prescribed by M.O.M. kit instructions. DAB staining for Pax7 (performed on freshly isolated, cytocentrifuged cells) was completed using the R.T.U. Vectastain Elite and ImmPact DAB substrate kits (both from Vector labs) following manufacturer's instructions. For X-gal staining, cells and tissue sections were fixed for 10 min at room temperature with 2% formaldehyde/0.2% glutaraldehyde and incubated overnight at 37°C with staining solution containing a final concentration of 1 mg/ml X-gal, 5 mM potassium ferricyonide, 5 mM potassium ferrocyonide, and 2 mM CaCl_2_ (all from Fisher). All stainings included negative controls in which primary antibodies or X-gal was omitted (depicted in Figure S4). Brightfield photographs were taken with Fisher Micromaster digital inverted microscope with Infinity optics using Micron v1.05 software. Immunofluorescent stainings were acquired with a Zeiss Axiovert 200 microscope equipped with monochrome camera. Controls for immunofluorescent stainings were taken at the same or greater exposure time as primary stained samples. Monochromatic photographs were colored then merged with Adobe Photoshop v9.0.2. When necessary to reduce background, certain photos' brightness and contrast levels were adjusted within individual channels prior to coloring. A list of primary and secondary antibodies, dilutions and microscope specifications are described in the Methods S1.

### Reverse-Transcription and Quantitative-RT-PCR

Methods and reagents for PCR reactions have been previously described by us [20]. Briefly RNA was purified from sorted cells using Qaigens RNeasy kit. All reactions were two-step beginning with cDNA synthesis followed by conventional or quantitative PCR for specific target genes. The following thermal cycling conditions were used: 95°C -7′ initial activation followed by 94°C-30″;57°C-30″;72°C-45″, for 35 cycles. Quantitative PCR reactions were run on an ABI 7900HT PCR system using sybr green PCR master mix. Results were analyzed using SDS 2.2 software and relative expressions calculated by comparative Ct method. cDNA from unsorted muscle mononuclear cells (Figure 1B) or whole muscle (Figures 4B and 6C) were used for calibration. The endogenous control was GAPDH. Ct values are listed in the Table S1. Each sample was derived from specific cell populations sorted from individual mice. Reactions for each respective sample and gene were run in triplicate. The relative expression between samples was used to calculate the average expression values and SEM for error bars represented in each figure. Primer sequences for each target gene are listed in Methods S1.

### Injury and Transplants

Muscle injury was induced by directly injecting 50 µl and 100 µl per TA and quadriceps, respectively, of 10 mM *Naja nigrcollis* cardiotoxin (Calbiochem) in PBS. Donor cells were sorted as CD45−/CD31−/Sca1−/α7+/CD34+ and CD34− from injured chicken actin promoter driven EGFP or *Myf5^nlacZ/+^* limb muscles injected with CTX 3 days prior to isolation. Cells were directly injected in 15–20 µl collection media, into uninjured or injured quadriceps treated with CTX the day prior to cell transplantation. For GFP cell analysis each recipients qaudricep (n = 12, 6 injured 1 day prior and 6 uninjured) was transplanted with 50,000 sorted CD34+/− cells. Recipients of GFP cells were euthanized 3 days post transplant and GFP donor cells were analyzed by FACS vs. uninjected injured and uninjured controls. Donor GFP cells represented <1% of all α7+ (CD45−/CD31−/Sca1−) cells. Quadriceps (n = 6, 3 injured/3 uninjured) injected with 10,000 *Myf5^nlacZ/+^* CD34− sorted cells were harvested 7 days post transplant and frozen in OCT for subsequent staining. For negative control, 50,000–150,000 sorted *Myf5^nlacZ/+^* (CD45−/CD31−/Sca1−) α7- cells (n = 2) were transplanted along with a single SHAM (media only) injection into uninjured quadriceps.

### BrdU Incorporation and Analysis

For each pulse 250 µl of 4 mg/ml BrdU (Sigma) in PBS was administered daily via intraperitoneal injection. CD34+/− cells were sorted from limb muscles, fixed with 4% formaldehyde for 5 minutes, washed with PBS and left overnight at 4°C. Tibialis and femur bone marrows were collected as a positive control for BrdU incorporation by flushing the tibia and femur with a 28 gauge needle. Fixed cells were processed and stained with PE conjugated anti-BrdU antibody (BD) according to manufacturer's protocol. Briefly cells were further fixed with 70% EtOH, denatured with 2 M HCL, and stained in PBS containing 0.5% Tween20 and 0.5% BSA. Sorted cells were identified as DAPI positive and BrdU incorporation was defined by comparing positive bone marrow cells with IgG isotype stained controls.

## Supporting Information

Methods S1 Includes antibodies and dilutions, fluorescent microscope specifications, antibody/fluorophore combinations used for each FACS experiment, BD flow cytometer specifications, and primers.
(0.12 MB DOC)

Figure S1 **FACS size selection strategy.** In order to remove duplets and debris we first gate on the tight population of small, low granulated events located at the bottom left of the Forward Scatter Area (FSC-A, x-axis) vs. Side Scatter Area (SSC-A, y-axis) graph. Next we select events by Forward Side Scatter Area (FSC-A, x-axis) vs. Width (FSC-W, y-axis) to remove the smallest and largest events. Finally we remove events that are too granulated by Side Scatter Area (SSC-A, x-axis) vs. Side Scatter Width (SSC-W, y-axis) then move to antigen selection depicted in Figure 5A. For single cell deposition DAPI negative cells (live cells) were selected prior to antigen selection (not shown).
(1.18 MB TIF)

Figure S2 **Following injury the majority of α7 integrin+ cells are negative for CD34, α-smooth muscle actin, and CD31.** Cryosection staining for α-smooth muscle actin (αSMA), CD31 and CD34 illustrates the majority of α7 integrin+ cells arising 3 days post CTX injury, are not endothelial or smooth muscle cells. (A) αSMA and CD31 staining shows the smooth muscle cells are confined to large vessels within injured muscle. (B) αSMA and α7 integrin staining confirms that the α7 integrin+ cells are not vascular smooth muscle cells within damaged areas of muscle (C) Staining for CD34 and α7 integrin shows the α7 integrin+ cells in the injured area are CD34-, while the majority of CD34+ cells represent vessels in the less injured region. Scale bars = 50 µm.
(5.21 MB TIF)

Figure S3 **X-gal staining reveals the presence CD34- *Myf5^nlacZ/+^* donor cells in CTX injured and uninjured quadriceps.** 10,000 sorted CD34- *Myf5^nlacZ/+^* cells were transplanted into CTX injured (n = 3) and uninjured (n = 3) quadriceps. Prior to immunostaining, X-gal was used to identify and confirmed the presence *Myf5^nlacZ/+^* donor cells. Scale bars = 50 µm.
(8.33 MB TIF)

Figure S4 **Staining controls.** Each figure's negative controls omitting primary antibodies or X-gal, positive control for X-gal, and negative transplant control. (A) Fig. 1C Pax7, CD34 and α7 integrin staining control. (C) Fig. 2 Pax7, laminin, and CD34 control. (B) Fig. 4A right panels; cultured Myf5 nlacZ/+ cells treated with stain solution containing and omitting x-gal. Fig. 4A right panels; cytocentrifuged sorted endothelial cells (CD45-/CD31+/Sca1+) stained with anti-Pax7 and unsorted mononuclear cells stained with secondary antibody as negative controls for Pax7 DAB staining. (D) CTX injured muscle staining control for Figures 7A and S2. (E) Fig. 8B&C β-gal and CD34 staining control. Far right panel portrays cryosection from (CD45-/CD31-/Sca1-) α7 integrin negative transplanted cells which stained negative for β-gal and positive for αSMA only in vascular smooth muscle cells. Scale bars = 50 µm.
(2.99 MB TIF)

Table S1 **q-RT-PCR average Ct values for each figure.**
(0.07 MB DOC)
