# Supplementary material for: Absence of CD34 on Murine Skeletal Muscle Satellite Cells Marks a Reversible State of Activation during Acute Injury
Source: PLoS One. 2010 Jun 2;5(6):e10920. doi: 10.1371/journal.pone.0010920 (PMC2880004; doi:10.1371/journal.pone.0010920)
Supplement: Methods S1 — Includes antibodies and dilutions, fluorescent microscope specifications, antibody/fluorophore combinations used for each FACS experiment, BD flow cytometer specifications, and primers. (0.12 MB DOC) [file pone.0010920.s001.doc]

**Supplementary Materials and Methods**

Antibodies and Dilutions

| **Histology Primary Antibodies** | | | | | |
| --- | --- | --- | --- | --- | --- |
| **Antigen** | **Conjugate** | **Host** | **Clone** | **Manufacturer** | **Dilution** |
| CD34 | Purified or Biotin | Rat | RAM34 | eBioscience | 1:200 |
| CD31 (PECAM1) | FITC | Rat | 390 | eBioscience | 1:50 |
| α7 integrin | FITC | Rat | 339408 | R&D | 1:100 |
| α7 integrin | Purified | Rat | 339408 | R&D | 1:200 |
| Laminin | Purified | Rabbit | Polyclonal | Sigma | 1:50 |
| Pax7 | Purified | Mouse | Pax7 | R&D | 1:50 |
| α-Smooth Muscle Actin | Cy3 or FITC | Mouse | 1A4 | Sigma | 1:500 |
| myogenin | Purified | Mouse | F5D | BD | 1:50 |
| β-galactosidase | Purified | Rabbit | Polyclonal | Invitrogen | 1:100 |
| **Histology Secondary Antibodies** | | | | | |
| Streptavidin | Alexa Fluor® 594 | n/a |  | Invitrogen | 1:800 |
| Streptavidin | Alexa Fluor® 647 | n/a |  | Invitrogen | 1:800 |
| Anti-Rat IgG | Alexa Fluor® 488 | Donkey |  | Invitrogen | 1:800 |
| Anti-Rabbit IgG | Alexa Fluor® 488 or 594 | Goat |  | Invitrogen | 1:800 |
| Anti-Mouse IgG (included with Vector M.O.M kit) | Biotin | Horse |  | Vector Labs | 1:250 |
| **FACS Antibodies and Streptavidin Conjugates** | | | | | |
| CD31 (PECAM-1) | PE-Cy7 | Rat | 390 | eBioscience | 3μl/10^6^ cells |
| CD45 (Ly-5) | PE-Cy5 or  Alexa Fluor® 700 | Rat | 30-F11 | eBioscience | 3μl/10^6^ cells |
| Sca1 (Ly-6 A/E) | APC or  eFluor™ 605 | Rat | D7 | eBioscience | 1.5μl/10^6^ cells |
| α7 integrin | FITC or APC | Rat | 339408 | R&D | 4μl/10^6^ cells |
| α7 integrin | FITC or PE | Mouse | 3C12 | MBL | 4μl/10^6^ cells |
| CD34 | Biotin | Rat | RAM34 | eBioscience | 3μl/10^6^ cells |
| Streptavidin | Pacific Blue | n/a |  | Invitrogen | 2μl/10^6^ cells |
| Streptavidin | PE-TexasRed | n/a |  | Invitrogen | 2μl/10^6^ cells |
| BrdU | PE | Mouse | 3D4 | BD | 10μl/sample <10^5^ cells |
| IgG Isotype | PE | Mouse | MOPC-21 | BD | 10μl/sample <10^5^ cells |
| DAPI |  |  |  | Sigma | F/C 10μg/ml |
| Note: FACS antibodies were mixed in a final volume of 100μl PBS with 0.3% BSA per 10^6^ cells for each staining. Following incubation with biotynilated anti-CD34 and wash, cells were incubated with a cocktail of anti-CD45, CD31, Sca1 and streptavidin. | | | | | |

Fluorescent Microscope Specifications

| Zeiss Axiovert 200 | |
| --- | --- |
| Monochrome Camera (onboard) | Axiocam mRM |
| Software | Axiovision v4.6.3 |
| Objectives | A-Plan 10x and 20x, Plan-Neofluar 40x |
| Power | HBO 100 - Mercury bulb |
| Manufactures mirror/filter set number, bandpass emission range, and detected fluorophores  (Note, all filters are bandpass) | 49, 420-470nm - DAPI  10, 515-565nm - FITC and Alexa Fluor® 488  43, 570-690nm - Cy3 and Alexa Fluor® 594  50, 665-715nm - Alexa Fluor® 647 |

**Antibody/Fluorophore Combinations Used for Each FACS Experiment**

| **Application** | **Aria I** | **Aria II** |
| --- | --- | --- |
| Pooled muscle analysis, cytocentrifugation, RNA and culture | CD45 PE-Cy5  CD31 PE-Cy7  Sca1 APC  CD34 Pacific Blue with  α7 integrin PE or FITC |  |
| Single Cell Deposition | CD45 PE-Cy5  CD31 PE-Cy7  Sca1 APC  CD34 PE-Texas Red  α7 FITC  DAPI |  |
| Individual Muscle and CTX injury Analysis | CD45 PE-Cy5  CD31 PE-Cy7  Sca1 APC  CD34 PE-Texas Red  α7 FITC |  |
| Cells sorted for BrdU staining (figure6B) |  | CD45 Alexa Fluor® 700  CD31 PE-Cy7  Sca1 eFluor™ 605  CD34 PE-Texas Red  α7 FITC |
| BrdU analysis of sorted cells following processing |  | DAPI  BrdU or IgG isotype PE |
| Isolation of donor cells for transplants and GFP analysis |  | CD45 Alexa Fluor® 700  CD31 PE-Cy7  Sca1 APC  CD34 Pacific Blue  α7 PE  GFP |
| Note: antibody cocktails reflect fluorophore combinations for optimal detection and minimal spillover with each respective Aria cell sorter. For the Aria I, Pacific Blue was used in place of PE-Texas Red to avoid spillover with PE. In addition, the Aria II yellow-green laser excited both APC and PE-Cy5 causing significant spillover whereas. Therefore on the Aria II we did not use PE-Cy5 in combination with APC but switched to Alexa Fluor® 700 for CD45. | | |

**BD Flow Cytometer Specifications**

|  | **FACSAria I** | **FACSAria II** |
| --- | --- | --- |
| Laser | Violet 407nm  Blue 488nm  Red 633nm | Violet 405nm  Blue 488nm  Red 633nm  Yellow-Green 561nm |
| Violet Laser Filters and detected Fluor/ Antigen | 450/50 Pacific Blue CD34 or DAPI | 450/50 - DAPI  605/40 - eFluor™ 605 Sca1 |
| Blue Laser Filters and detected Fluor/ Antigen | 488/10 - FSC size/SSC granulation  530/30 - FITC α7 integrin  575/26 - PE α7 integrin  610/20 - PE-Texas Red CD34  675/20 - PE-Cy5 CD45  780/60 - PE-Cy7 CD31 | 488/10 - FSC size/SSC granulation  535/30 - FITC α7 integrin or GFP |
| Red Laser Filters and detected Fluor/ Antigen | 660/20 - APC Sca1 | 670/30 - APC α7 integrin  730/45 - Alexa Fluor® 700 CD45 |
| Yellow-Green Laser Filters and detected Fluor/ Antigen | n/a | 780/60 - PE-Cy7 CD31 |
| Software | FACSDiva™ v6.0 or later | |
| Compensation | For every experiment, unstained and single antibody/color stained controls were used for software automated compensation. | |
| Data Conversion File Format | FCS 3.0 | |
| Note: Fluorescent emission is directed by longpass mirrors (not listed) into specified bandpass filters. | | |

**Primers**

| **RT-PCR Primers** | | |
| --- | --- | --- |
| **Target Gene** | **Forward Primer** | **Reverse Primer** |
| *Tie1* | TGTTCGTGGCCTCAATGCTA | TCGGATACACACCAAGGCTAA A |
| *vWF* | GATGTCCAGCTCCCCTTCCT | AGGCGTTTCCGAAGTCTACCA |
| *GAPDH* | CTCGTCCCGTAGACAAAATGG | CGCTCCTGGAAGATGGTG |
| **Quantitative RT-PCR Primers** | | |
| **Target Gene** | **Forward Primer** | **Reverse Primer** |
| *α7 integrin* | GCCCCCCGGAATCTACTATCT | CGATAGCCGGTGGTAAGAACA |
| *CD34* | CGCAGTTGGAGCCCTACAG | CCTCCACCATTCTCCGTGTAAT |
| *Pax3* | TCGGCCTTGCGTCATTTC | CAGGATCTTAGAGACGCAACCA |
| *Pax7* | GCTGCTGAAGGACGGTCACT | TCGATGCTGTGTTTGGCTTTC |
| *Myf5* | CCAGCCCCACCTCCAACT | GGGACCAGACAGGGCTGTTA |
| *MyoD* | CACTCCGGGACATAGACTTGACA | TCGAAACACGGATCATCATAGAA |
| *myogenin* | CCCATGGTGCCCAGTGAA | GCAGATTGTGGGCGTCTGTA |
| *CXCR4* | GCCCTCGCCTTCTTCCA | CCCCGAGGAAGGCATAGAG |
| *c-met* | GCAGTGACGAGTATCGGACAGA | GCCGGCCCATGAATAAGTC |
| *NCAM* | CAACATGGAGGGCATTGTCA | CGATCCGAGTACGTCGTCTCA |
| *GAPDH* | GGGAAGCCCATCACCATCT | GCCTCACCCCATTTGATGTT |
