# Supplementary figures and images for: Absence of CD34 on Murine Skeletal Muscle Satellite Cells Marks a Reversible State of Activation during Acute Injury
Source: PLoS One. 2010 Jun 2;5(6):e10920. doi: 10.1371/journal.pone.0010920 (PMC2880004; doi:10.1371/journal.pone.0010920)

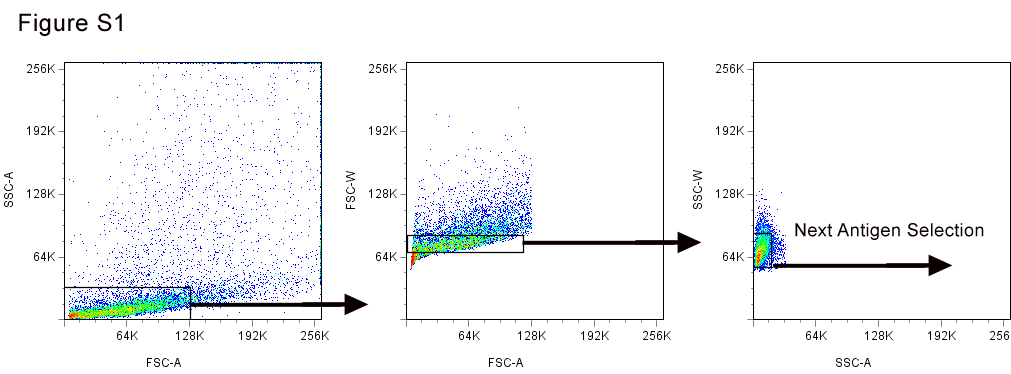

Supplement: Figure S1 — FACS size selection strategy. In order to remove duplets and debris we first gate on the tight population of small, low granulated events located at the bottom left of the Forward Scatter Area (FSC-A, x-axis) vs. Side Scatter Area (SSC-A, y-axis) graph. Next we select events by Forward Side Scatter Area (FSC-A, x-axis) vs. Width (FSC-W, y-axis) to remove the smallest and largest events. Finally we remove events that are too granulated by Side Scatter Area (SSC-A, x-axis) vs. Side Scatter Width (SSC-W, y-axis) then move to antigen selection depicted in Figure 5A. For single cell deposition DAPI negative cells (live cells) were selected prior to antigen selection (not shown). (1.18 MB TIF) [file pone.0010920.s002.tif]

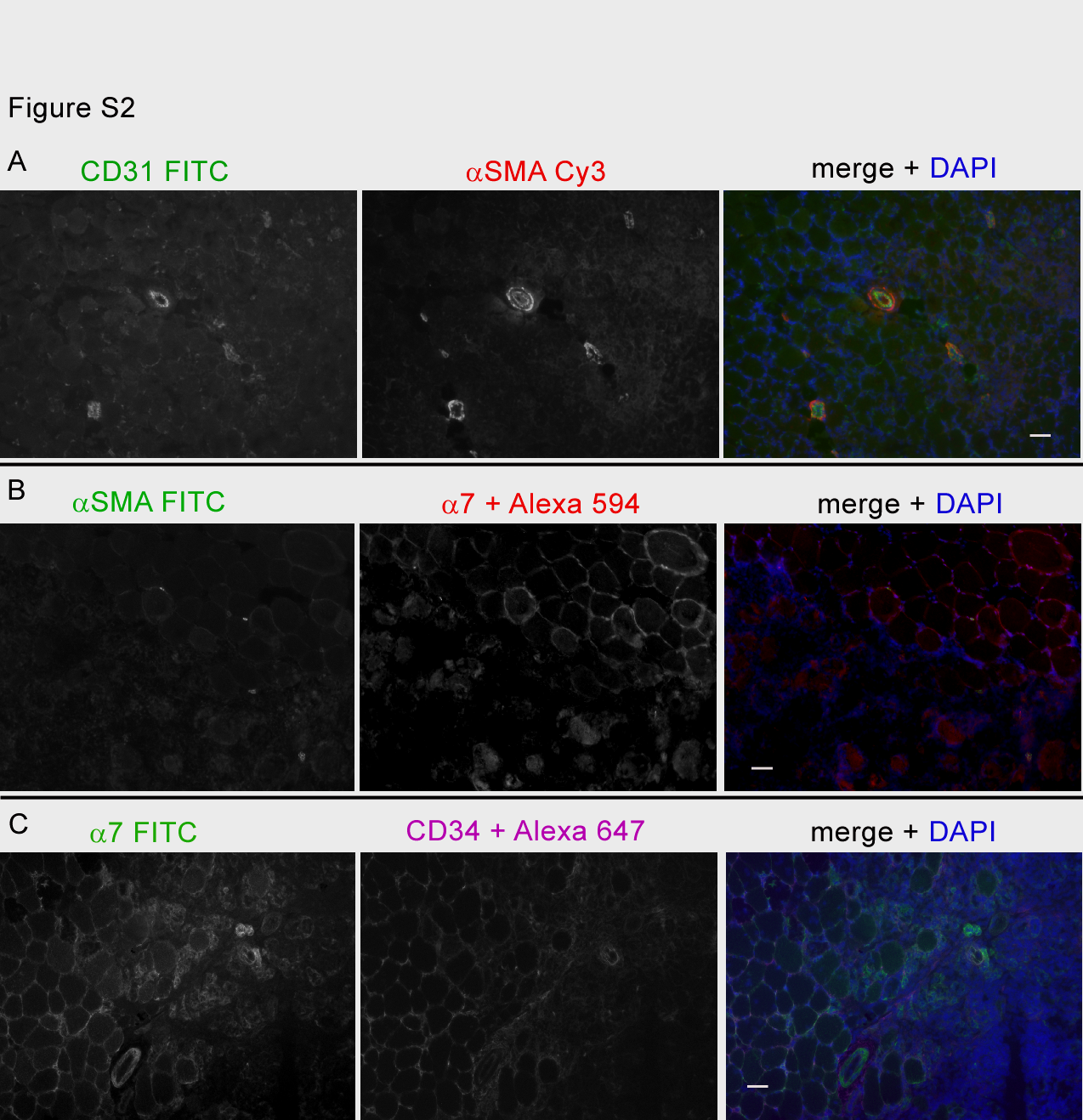

Supplement: Figure S2 — Following injury the majority of α7 integrin+ cells are negative for CD34, α-smooth muscle actin, and CD31. Cryosection staining for α-smooth muscle actin (αSMA), CD31 and CD34 illustrates the majority of α7 integrin+ cells arising 3 days post CTX injury, are not endothelial or smooth muscle cells. (A) αSMA and CD31 staining shows the smooth muscle cells are confined to large vessels within injured muscle. (B) αSMA and α7 integrin staining confirms that the α7 integrin+ cells are not vascular smooth muscle cells within damaged areas of muscle (C) Staining for CD34 and α7 integrin shows the α7 integrin+ cells in the injured area are CD34-, while the majority of CD34+ cells represent vessels in the less injured region. Scale bars = 50 µm. (5.21 MB TIF) [file pone.0010920.s003.tif]

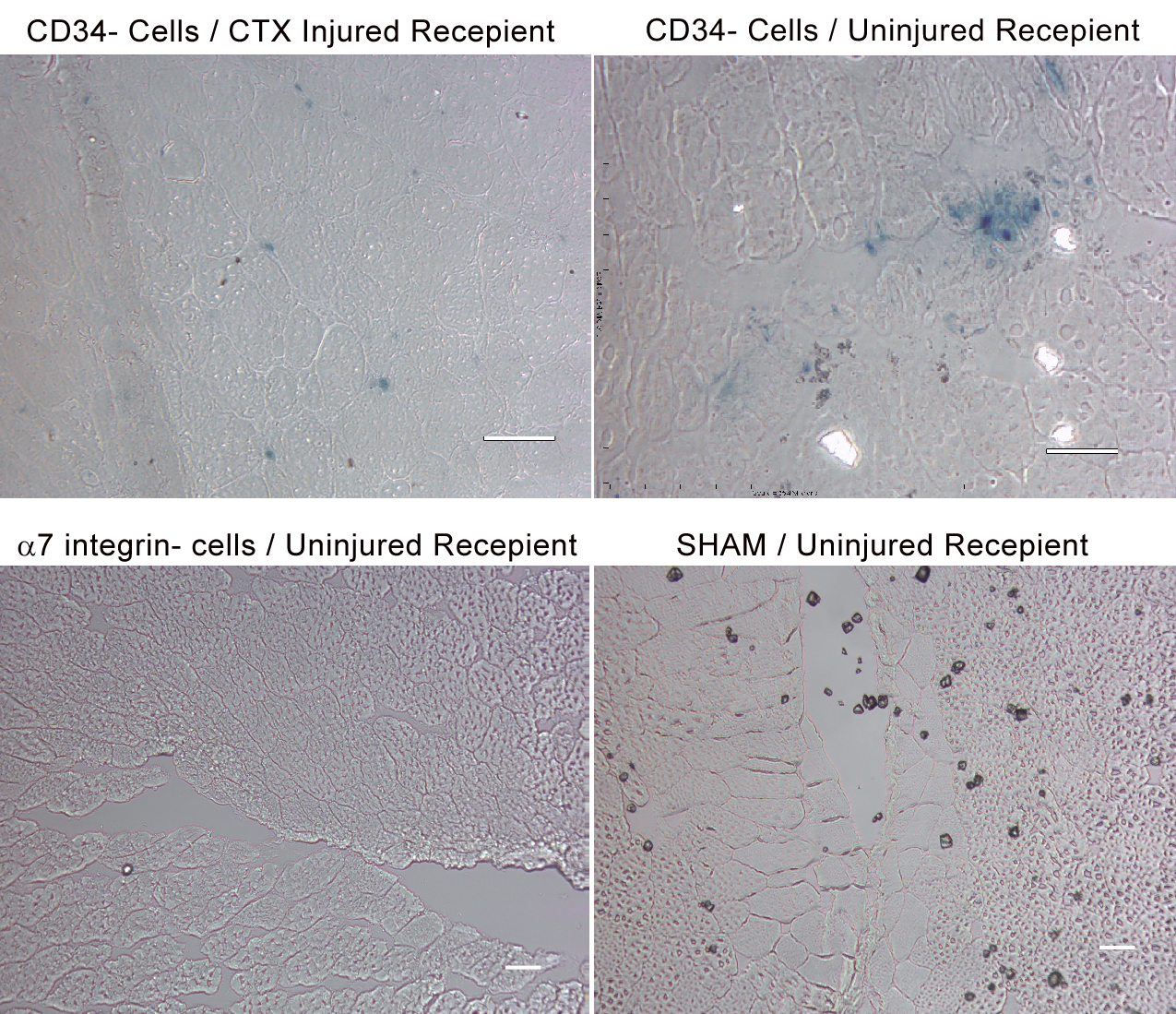

Supplement: Figure S3 — X-gal staining reveals the presence CD34- Myf5nlacZ/+ donor cells in CTX injured and uninjured quadriceps. 10,000 sorted CD34- Myf5nlacZ/+ cells were transplanted into CTX injured (n = 3) and uninjured (n = 3) quadriceps. Prior to immunostaining, X-gal was used to identify and confirmed the presence Myf5nlacZ/+ donor cells. Scale bars = 50 µm. (8.33 MB TIF) [file pone.0010920.s004.tif]

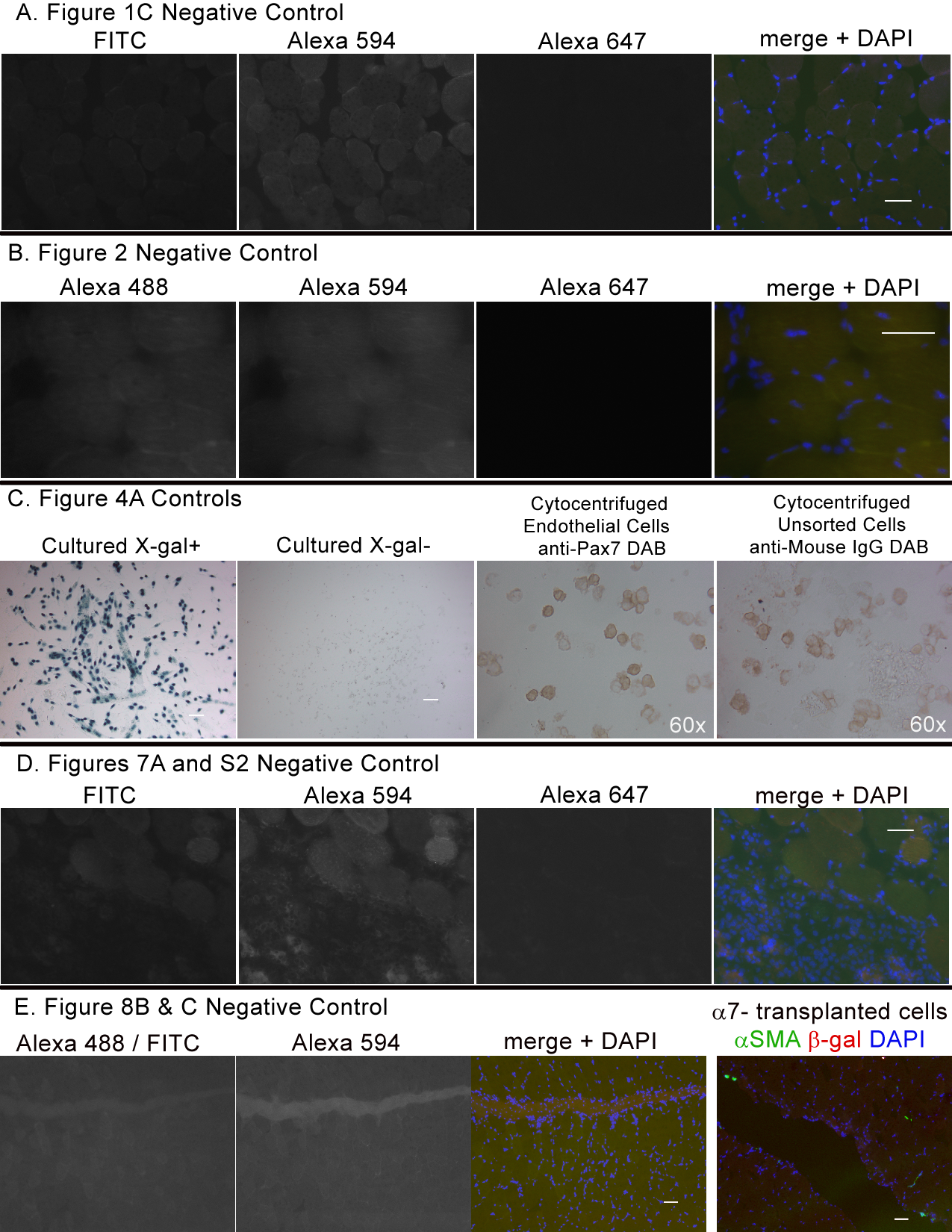

Supplement: Figure S4 — Staining controls. Each figure's negative controls omitting primary antibodies or X-gal, positive control for X-gal, and negative transplant control. (A) Fig. 1C Pax7, CD34 and α7 integrin staining control. (C) Fig. 2 Pax7, laminin, and CD34 control. (B) Fig. 4A right panels; cultured Myf5 nlacZ/+ cells treated with stain solution containing and omitting x-gal. Fig. 4A right panels; cytocentrifuged sorted endothelial cells (CD45-/CD31+/Sca1+) stained with anti-Pax7 and unsorted mononuclear cells stained with secondary antibody as negative controls for Pax7 DAB staining. (D) CTX injured muscle staining control for Figures 7A and S2. (E) Fig. 8B&C β-gal and CD34 staining control. Far right panel portrays cryosection from (CD45-/CD31-/Sca1-) α7 integrin negative transplanted cells which stained negative for β-gal and positive for αSMA only in vascular smooth muscle cells. Scale bars = 50 µm. (2.99 MB TIF) [file pone.0010920.s005.tif]
