# Supplementary material for: Absence of CD34 on Murine Skeletal Muscle Satellite Cells Marks a Reversible State of Activation during Acute Injury
Source: PLoS One. 2010 Jun 2;5(6):e10920. doi: 10.1371/journal.pone.0010920 (PMC2880004; doi:10.1371/journal.pone.0010920)
Supplement: Table S1 — q-RT-PCR average Ct values for each figure. (0.07 MB DOC) [file pone.0010920.s006.doc]

**Table S1. q-RT-PCR Average Ct Values for each Figure.**

| **Figure 1C** | | | | |
| --- | --- | --- | --- | --- |
|  | **CD34+/α7+ cells** | | **CD34+/α7- cells** | |
|  | **Sample1** | **Sample2** | **Sample1** | **Sample2** |
| *Pax7* | 31.88 | 29.15 | 38.23 | 38.35 |
| *Myf5* | 30.92 | 28.32 | 36.41 | 37.08 |
| *GAPDH* | 29.62 | 26.88 | 29.50 | 30.11 |

| **Figure 2C** | | | | | | |
| --- | --- | --- | --- | --- | --- | --- |
|  | **CD34+** | | | **CD34-** | | |
|  | **Sample1** | **Sample2** | **Sample3** | **Sample1** | **Sample2** | **Sample3** |
| *α7 integrin* | 27.13 | 25.65 | 27.74 | 26.19 | 25.85 | 25.92 |
| *CD34* | 28.01 | 27.20 | 28.52 | 32.69 | 33.03 | 32.36 |
| *Pax3* | 29.85 | 30.94 | 30.75 | 33.36 | 34.64 | 33.54 |
| *Pax7* | 25.74 | 24.16 | 25.95 | 29.65 | 30.33 | 28.74 |
| *Myf5* | 26.29 | 24.83 | 25.19 | 29.07 | 29.58 | 29.32 |
| *MyoD* | 31.99 | 30.28 | 30.45 | 33.62 | 33.86 | 34.33 |
| *myogenin* | 33.24 | 30.60 | 32.23 | 40.00 | 40.00 | 36.79 |
| *GAPDH* | 24.02 | 23.03 | 23.98 | 24.46 | 23.83 | 22.98 |
|  | | | | | | |
| *CXCR4* | 27.18 | 25.76 | 27.03 | 30.78 | 30.61 | 29.55 |
| *c-met* | 30.85 | 29.55 | 31.15 | 33.99 | 33.65 | 32.88 |
| *NCAM* | 33.98 | 31.98 | 33.78 | 35.47 | 36.36 | 34.56 |
| *GAPDH* | 26.34 | 24.96 | 26.41 | 25.61 | 25.27 | 25.49 |

| **Figure 5C** | | | | | | |
| --- | --- | --- | --- | --- | --- | --- |
|  | **CD34+ CTX injured** | | | **CD34- CTX injured** | | |
|  | **Sample1** | **Sample2** | **Sample3** | **Sample1** | **Sample2** | **Sample3** |
| *Myf5* | 25.96 | 26.60 | 27.48 | 24.19 | 25.11 | 24.25 |
| *MyoD* | 26.85 | 28.30 | 29.24 | 25.55 | 27.85 | 25.18 |
| *myogenin* | 26.45 | 27.65 | 28.39 | 22.93 | 25.26 | 24.28 |
| *GAPDH* | 21.42 | 22.69 | 23.26 | 19.32 | 21.36 | 20.87 |
